# Dithieno[3,2-*b*:2′,3′-*d*]pyridin-5(4*H*)-one based D–A type copolymers with wide bandgaps of up to 2.05 eV to achieve solar cell efficiencies of up to 7.33%†
†Electronic supplementary information (ESI) available: TGA plots, DFT calculations, device structure, ^1^H NMR of polymers and mean value of key parameters of PCEs. See DOI: 10.1039/c6sc01791f


**DOI:** 10.1039/c6sc01791f

**Published:** 2016-06-10

**Authors:** Wei Gao, Tao Liu, Minghui Hao, Kailong Wu, Chen Zhang, Yanming Sun, Chuluo Yang

**Affiliations:** a Hubei Collaborative Innovation Center for Advanced Organic Chemical Materials , Hubei Key Lab on Organic and Polymeric Optoelectronic Materials , Department of Chemistry , Wuhan University , Wuhan 40072 , China . Email: clyang@whu.edu.cn; b Heeger Beijing Research and Development Center , School of Chemistry and Environment , Beihang University , Beijing 100191 , P. R. China . Email: sunym@buaa.edu.cn

## Abstract

Two new polymers, **PDTPO-IDT** and **PDTPO-IDTT**, are synthesized through copolymerization of 4-(2-octyldodecyl)-dithieno[3,2-*b*:2′,3′-*d*]pyridin-5(4*H*)-one (**DTPO**) with indacenodithiophene (**IDT**) or indacenodithieno[3,2-*b*]thiophene (**IDTT**). The rational combination of the planar **DTPO** unit with ladder-type **IDT** and **IDTT** units endows the resulting copolymers with wide optical bandgaps of *ca.* 2.05 eV, low HOMO energy levels of *ca.* –5.32 eV and good hole-transporting abilities with a hole mobility of 1.0 × 10^–3^ cm^2^ V^–1^ s^–1^. The polymer solar cell (PSC) in a conventional structure based on **PDTPO-IDT** as donor and [6,6]-phenyl-C_71_-butyric acid methyl ester (PC_71_BM) as acceptor achieves a high power conversion efficiency (PCE) of up to 7.33%, the highest value for PSCs based on polymers with optical bandgap over 2.0 eV to date, along with a remarkable open-circuit voltage (*V*_oc_) approaching 0.97 V. The performance of the **PDTPO-IDTT** based PSC is slightly behind this with a moderate PCE of 5.47% under the same conditions. The relationship between the copolymer structures and optoelectronic properties as well as photovoltaic performance are comprehensively investigated by experiments and theoretical simulations.

## Abstract 2

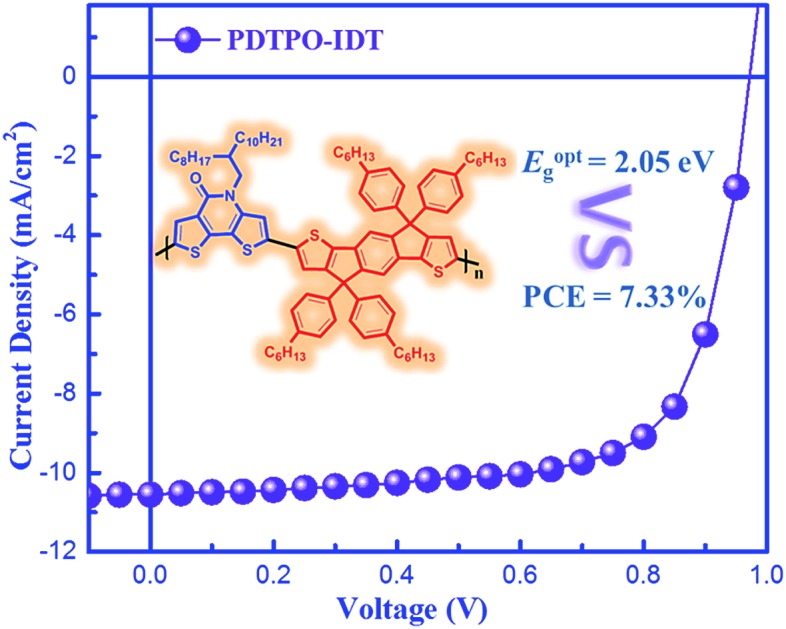
A PCE of 7.33% was achieved in a PSC based on a new copolymer, **PDTPO-IDT**, with bandgaps of up to 2.05 eV.

## Introduction

Organic photovoltaics (OPVs), emerging as an environmentally benign technology with the function of converting solar energy into electricity, have shown attractive prospects owing to their low-cost, low weight, large area and flexible fabrication.^1^–^8^ Gratifyingly, through in-depth research on theories^9^–^11^ and novel materials,^5^,^12^–^16^ the power conversion efficiency (PCE) of bulk heterojunction (BHJ) solar cells based on conjugated polymers as donors and [6,6]-phenyl-C_71_/C_61_-butyric acid methyl ester (PC_71_BM or PC_61_BM) as acceptors have reached over 10% for single junction solar cells^17^–^22^ and 11% for tandem solar cells.^23^,^24^ Such high efficiency polymer solar cells (PSCs) usually employ photoactive materials with low or medium bandgaps, for the purpose of enlarging incident light harvesting and originally promoting short circuit current density (*J*_sc_). Compared to low and medium bandgap materials, wide bandgap materials have lagged behind, and only a few of them have achieved efficiencies of over 7%,^25^,^26^ in particular when the bandgap exceeds 2.0 eV. This is because such kinds of polymers suffer from an inherent defect of a narrow absorption band below 650 nm (optical bandgap [*E*_g_] > 1.9 eV) in the solar spectrum, which is not conducive to the full use of a high luminous flux section of the solar spectrum and directly limits the photocurrent at the first step of exciton generation. However, *J*_sc_ is only one of the key factors determining the efficiency of BHJ solar cells. That means we are able to optimize the other two key factors: open circuit voltage (*V*_oc_) and fill factor (FF) through reasonable material design and careful device processing. Moreover, from a material engineering perspective, *J*_sc_ can be fine-tuned by effective exciton separation and enhancement of charge-carrier mobility through morphological control, device structure optimization as well as interlayer engineering. In addition, wide bandgap materials are critical to construct high performance tandem solar cells in which they can act as a photoactive layer in the front cell of a tandem solar cell combined with a rear cell using low bandgap materials to implement the full absorption of the solar spectrum.^27^,^28^

Ladder-type electron-donor units with linearly fused aromatic or heteroaromatic subunits have been widely utilized to build low and medium bandgap materials.^29^–^32^ This is because covalently forced planarization can extend effective conjugation length along a polymer backbone and promote π-electron delocalization between parallel p-orbitals. Moreover, a structure with rigid coplanarity may restrain rotational disorder to lower reorganization energy, which benefits the charge carrier mobility. Indacenodithiophene (**IDT**) and indacenodithieno[3,2-*b*]thiophene (**IDTT**) are popular ladder-type building blocks with five-membered and seven-membered multifused rings, respectively. When they are copolymerized with common electron-deficient units, such as 4,7-dibromo-2,1,3-benzothiadiazole (**BT**),^31^,^33^–^35^ 4,7-dibromo-5,6-difluoro-2,1,3-benzothiadiazole (**2FBT**),^34^–^36^ 1,3-dibromo-thieno[3,4-*c*]pyrrol-4,6-dione (**TPD**),^35^,^37^–^39^ the absorption spectra of the resulting polymers often exhibit, to a certain extent, blue shifting compared with polymers based on 4,8-bis(2-ethylhexyloxy)benzo[1,2-*b*:4,5-*b*′]dithiophene (**BDTO**) and 4,8-bis(5-(2-ethylhexyl)thiophen-2-yl)benzo[1,2-*b*:4,5-*b*′]dithiophene (**BDTT**) coupled with similar acceptor units. This means that both **IDT** and **IDTT** are weaker electron donors than benzodithiophenes (**BDTO** and **BDTT**), which will facilitate wide bandgap.

Recently, Yu’s group and our group reported a novel structure of two thiophenes flanking a tricyclic pyridone, 4-(2-octyldodecyl)-dithieno[3,2-*b*:2′,3′-*d*]pyridin-5(4*H*)-one (**DTPO**).^40^,^41^ The copolymers based on **DTPO** and **BDTs** (**PDTPO-BDTO** and **PDTPO-BDTT**) showed bandgaps of around 2.0 eV and a relatively low HOMO energy level of –5.44 eV which led to a high *V*_oc_ of 0.98 V, and PCEs of up to 6.84% were achieved in PSCs. Meanwhile, the homopolymer of **DTPO** revealed a high hole mobility in an organic field-effect transistor (OFET). These results indicated that **DTPO** is a good building block for wide bandgap materials.

**Scheme 1:**
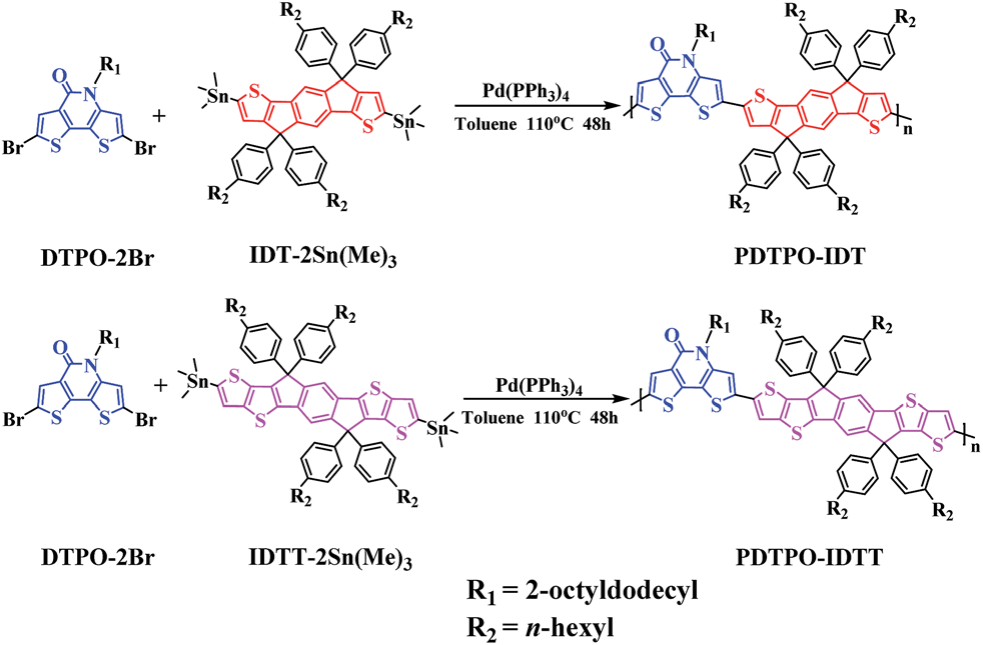
Synthesis and chemical structures of **PDTPO-IDT** and **PDTPO-IDTT**.

Aiming to explore new wide bandgap materials, herein, we designed and synthesized two new polymers, **PDTPO-IDT** and **PDTPO-IDTT** (Scheme 1), by replacing **BDTs** with **IDT** and **IDTT** units as electron donors to copolymerize with the **DTPO** unit. We anticipate that the combination of ladder-type **IDT** and **IDTT** units with the **DTPO** unit would facilitate the planarization of the copolymers, strengthen intermolecular π–π stacking and improve their charge-carrier transporting properties. The two copolymers show wide bandgaps of *ca.* 2.05 eV. Noticeably, PSCs based on **PDTPO-IDT** exhibited high *V*_oc_ of up to 0.98 V and achieved PCEs of up to 7.33%.

## Results and discussion

### Synthesis and characterization

As shown in Scheme 1, the copolymers of **PDTPO-IDT** and **PDTPO-IDTT** were prepared by the Stille polycondensation between the monomer of **DTPO-2Br** and commercially available monomers of **IDT-2Sn(Me)_3_** and **IDTT-2Sn(Me)_3_**. The structures of the two polymers were characterized by ^1^H NMR and elemental analysis (EA). The number-average molecular weights (*M*_n_) and polydispersity indices (PDI) of **PDTPO-IDT** and **PDTPO-IDTT** are 23.9 kDa/1.75 and 30.2 kDa/2.28, respectively, measured by gel permeation chromatography (GPC) with polystyrene as the standard. The two polymers showed good solubility in common solvents, such as chloroform (CF), tetrahydrofuran (THF), chlorobenzene (CB) and *o*-dichlorobenzene (*o*-DCB).

As shown in Fig. S1,† the decomposition temperatures (*T*_d_) with 5% weight loss are 440 °C for **PDTPO-IDT** and 436 °C for **PDTPO-IDTT**, indicating that the two polymers are stable enough for application in PSC devices.

### Optical properties

**Fig. 1:**
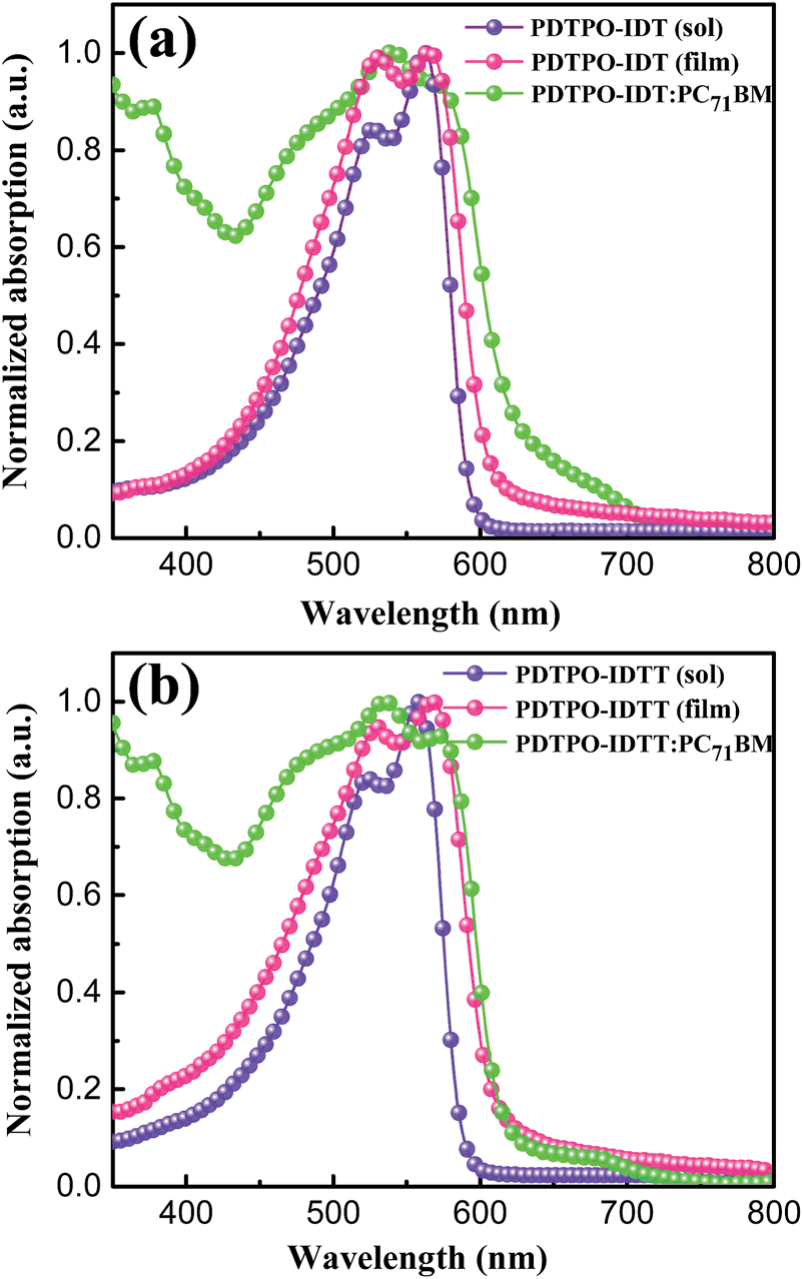
Normalized UV-vis absorption spectra in chloroform solution, pure films and blend films for **PDTPO-IDT** (a) and **PDTPO-IDTT** (b).

The UV-vis absorption spectra of **PDTPO-IDT** and **PDTPO-IDTT** in chloroform solution, pure films and blend films are shown in Fig. 1, and the corresponding absorption maxima and molar extinction coefficients are listed in Table 1. As shown, both the spectra profiles and absorption peaks of the two polymers are similar in chloroform solution and pure films, attributed to the similar structures between **IDT** and **IDTT**. Unlike the absorption spectra of other conjugated polymers with extended fused ring systems, the absorption peak resulting from localized π–π* transitions at short wavelengths is absent in both solution and pure films, which is similar to those polymers with optical bandgap over 2.0 eV.^25^,^43^–^45^ The broad absorption bands ranging from 400 to 600 nm are ascribed to intramolecular charge transfer (ICT). In addition, strong vibronic shoulder peaks are observed in both solution and pure films of the two polymers, demonstrating the existence of strong π–π stacking between polymer chains. The two polymers exhibit high molar extinction coefficients of 8.33 × 10^4^ M^–1^ cm^–1^ for **PDTPO-IDT** and 1.32 × 10^5^ M^–1^ cm^–1^ for **PDTPO-IDTT** in chloroform solution. In order to understand the absorption behavior of the active layers, the blend films of the polymers and PC_71_BM were investigated. The complementary absorption spectra of PC_71_BM and **PDTPO-IDT** contribute to a 24 nm expansion of the absorption band of **PDTPO-IDT**. In contrast, the spectra of PC_71_BM and **PDTPO-IDTT** blend films have a negligible effect on the absorption band at long wavelength. The optical bandgaps calculated from the absorption edge of pure films spectra are 2.05 eV for **PDTPO-IDT** and 2.04 eV for **PDTPO-IDTT**, respectively.

**Table 1:** Basic properties of **PDTPO-IDT** and **PDTPO-IDTT**

Polymer	*M*_n_ ^*a*^ (kDa)	PDI	*T*_d_ ^*b*^ (°C)	*ε*_max_ ^*c*^ (M^–1^ cm^–1^)	*λ*_max_ ^*c*^ (nm)	*λ*_max_ ^*d*^ (nm)	*λ*_onset_ ^*d*^ (nm)	*E* opt g ^*e*^ (eV)	HOMO (eV)	LUMO^*f*^ (eV)
**PDTPO-IDT**	23.9	1.7	440	8.33 × 10^4^	528	531	604	2.05	–5.32	–3.27
					563	566				
**PDTPO-IDTT**	30.2	2.2	436	1.32 × 10^5^	524	532	607	2.04	–5.31	–3.27
					557	569				

^*a*^Measured by GPC with polystyrene as the standard.

^*b*^Obtained from TGA with 5% weight loss.

^*c*^In chloroform solution.

^*d*^In pure film drop-cast from chloroform solution.

^*e*^Calculated from *E*optg = 1240/*λ*_onset_.

^*f*^Obtained from *E*_LUMO_ = *E*optg + *E*_HOMO_.

### Electrochemical properties

**Fig. 2:**
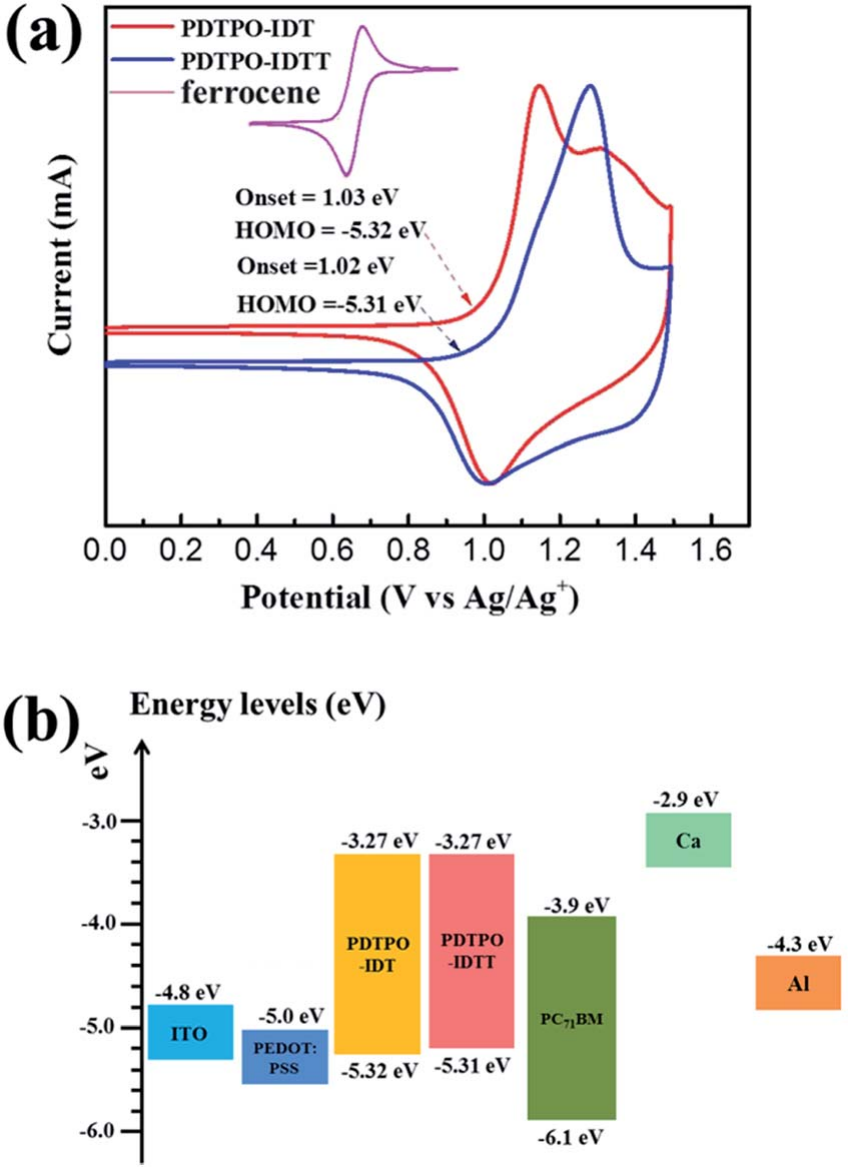
(a) The electrochemical cyclic voltammetry measurements of **PDTPO-IDT** and **PDTPO-IDTT** thin film coated on the platinum electrode in acetonitrile solution containing 0.1 M *n*-Bu_4_NPF_6_ at a scan rate of 100 mV s^–1^. (b) Schematic energy level diagram of all components used in conventional devices.

Cyclic voltammetry (CV) was carried out to estimate the highest occupied molecular orbital (HOMO) and lowest unoccupied molecular orbital (LUMO) energy levels of the two polymers. As displayed in Fig. 2a, both polymers show reversible oxidation processes. The oxidation onset potentials (*E*_ox_) of **PDTPO-IDT** and **PDTPO-IDTT** referenced to Ag/AgCl (Ag/Ag^+^) were found to be 1.03 V and 1.02 V, respectively, corresponding to HOMO energy levels of –5.32 eV and –5.31 eV. The LUMO energy levels were calculated from the equation: *E*_LUMO_ = (*E*_HOMO_ + *E*optg) eV. Both copolymers have the same LUMOs of –3.27 eV, which are higher than those of **PDTPO-BDTO** (–3.37 eV) and **PDTPO-BDTT** (–3.35 eV) which we previously reported.^40^ This means that the offsets between the LUMO energy levels of donor and acceptor (PC_71_BM) tend to become larger, which will provide a greater driving force to accelerate the exciton separation, and decrease the exciton recombination rate around the interface between donor and acceptor.

### Theoretical calculations

**Fig. 3:**
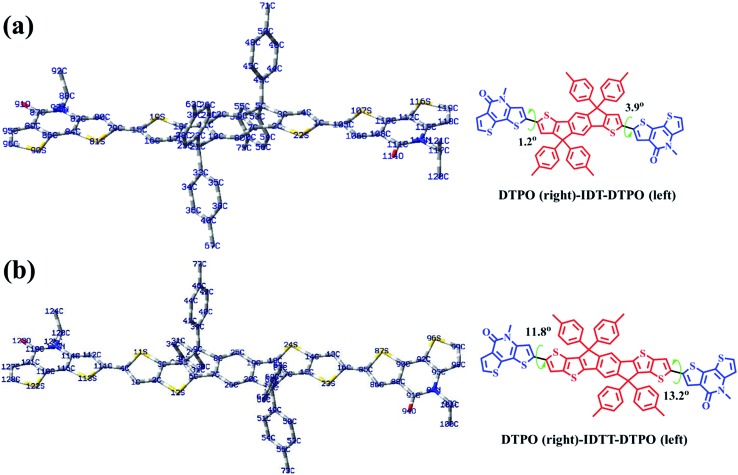
Theoretically calculated model molecules and corresponding torsion angles, and optimized conformations of three units of **PDTPO-IDT** (a) and **PDTPO-IDTT** (b) at the B3LYP/6-31G* level. Gray: carbon (C); blue: nitrogen (N); red: oxygen (O) and yellow: sulfur (S). Hydrogen atoms have been removed for clarity.

To further understand the effect of copolymer planarization, theoretical calculations were performed by using density functional theory (DFT) at the B3LYP/6-31G* level. Taking the asymmetrical characteristic of **DTPO** units into consideration, one and a half repeating units were employed as a simplified model to simulate the whole molecular skeleton. The long alkyl side chains were replaced with methyl groups to further simplify the calculation. As illustrated in Fig. 3, **DTPO** and **IDT**/**IDTT** units were found to be oriented in antiparallel equilibrium geometries. Due to the different chemical environments of C79 (C114) and C105 (C85) in the **DTPO** unit observed from the ^13^C NMR of **DTPO** (Fig. S5†), there is a 2.7° difference between the dihedral angle *φ*(C80–C79–C15–S19) (1.2°) and *φ*(C4–C1–C105–S107) (3.9°). A similar situation occurs for **DTPO-IDTT-DTPO** with the difference between the dihedral angles *φ*(C112–C111–C4–S11) (11.8°) and *φ*(C13–C16–C85–S87) (13.2°) being 1.4°. A larger torsion angle in **PDTPO-IDTT** results from larger steric hindrance of **IDTT** due to the interactions between ipsilateral alkyl chains of the donor and acceptor. The larger dihedral angles of **PDTPO-IDTT** over **PDTPO-IDT** suggest a larger change of the planarity from solution to solid state,^42^ which is consistent with a larger red shift of absorption spectra of **PDTPO-IDTT** from solution to solid state.

The HOMO and LUMO orbitals of the model compounds are shown in Fig. S2.† Both HOMO and LUMO frontier orbitals of **PDTPO-IDT** and **PDTPO-IDTT** are well-proportionally distributed along the whole conjugated plane, which would be favorable to the charge carrier transport. Unlike low bandgap polymers, such an orbital distribution reveals a weak intramolecular charge transfer, which explains the reason why the maximum absorption values of the two polymers are below 600 nm.

### Photovoltaic performance

**Fig. 4:**
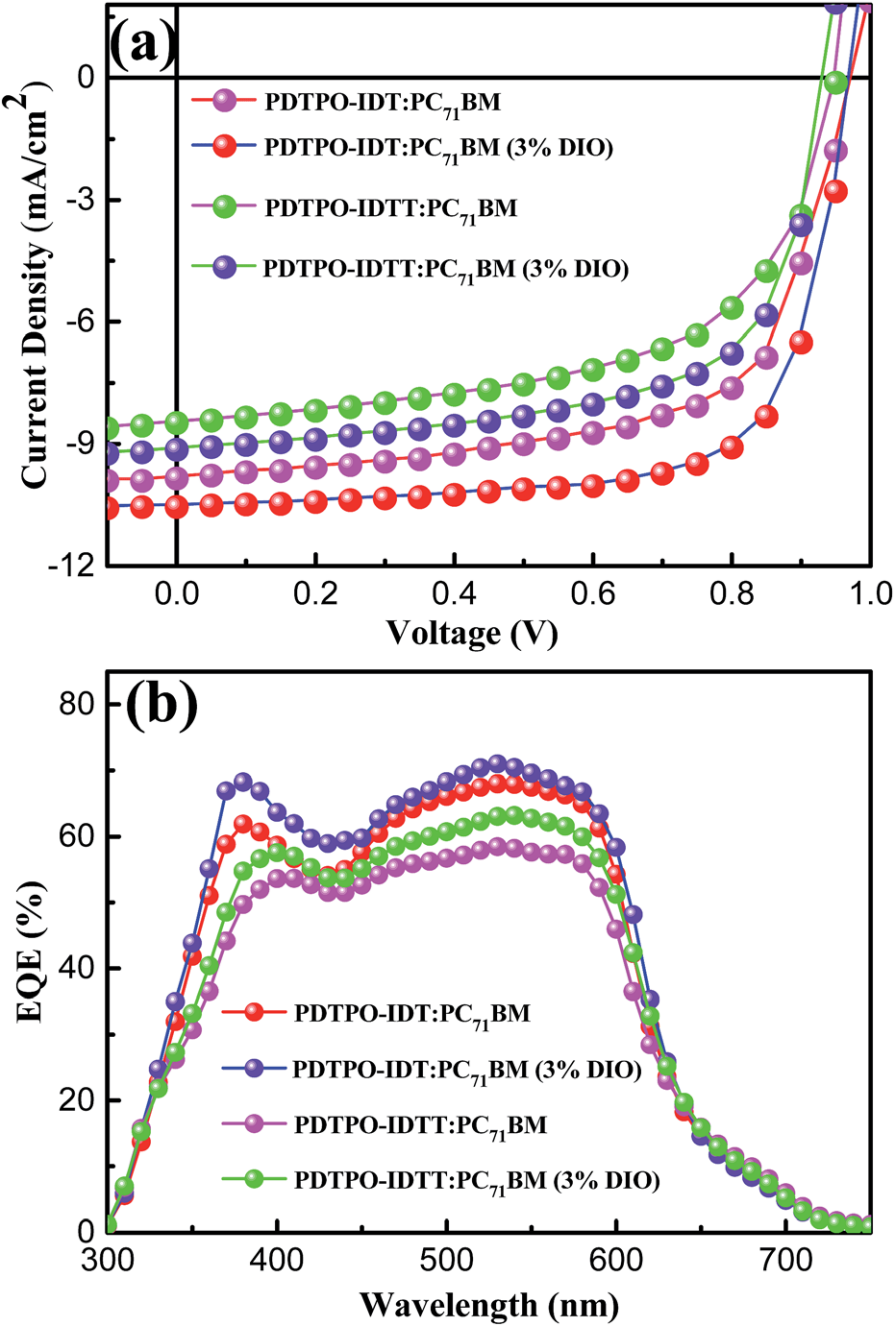
(a) *J*–*V* characteristics curves of **PDTPO-IDT** : PC_71_BM (1 : 2, w/w) and **PDTPO-IDTT** : PC_71_BM (1 : 2, w/w) based PSCs without or with 3% DIO (v/v) under AM 1.5 G at 100 mW cm^–2^. (b) Corresponding EQE spectra of **PDTPO-IDT** : PC_71_BM and **PDTPO-IDTT** : PC_71_BM based PSCs without or with 3% DIO (v/v).

**Fig. 5:**
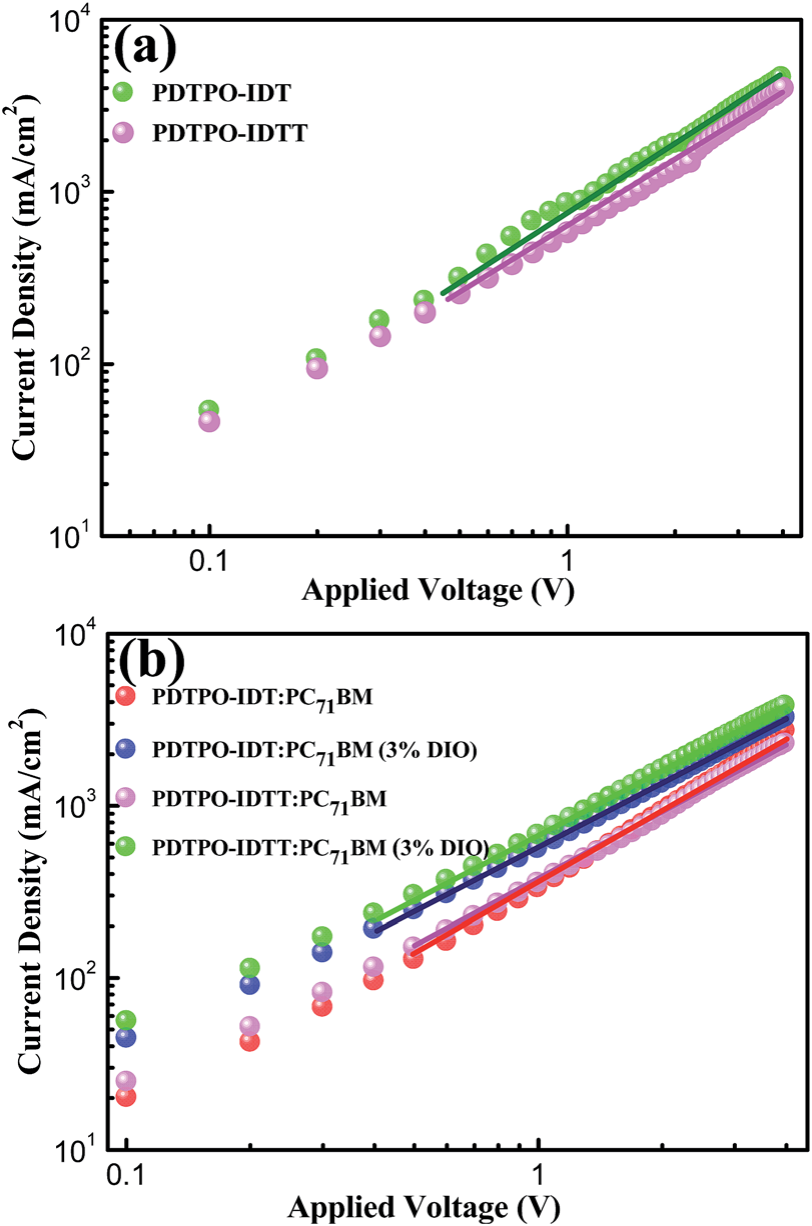
Current–voltage (*I*–*V*) characteristics of pure films (a) and blend films (b) (with or without 3% DIO) of **PDTPO-IDT** : PC_71_BM (1 : 2, w/w) and **PDTPO-IDTT** : PC_71_BM (1 : 2, w/w) in space-charge-limited current (SCLC) devices.

**Fig. 6:**
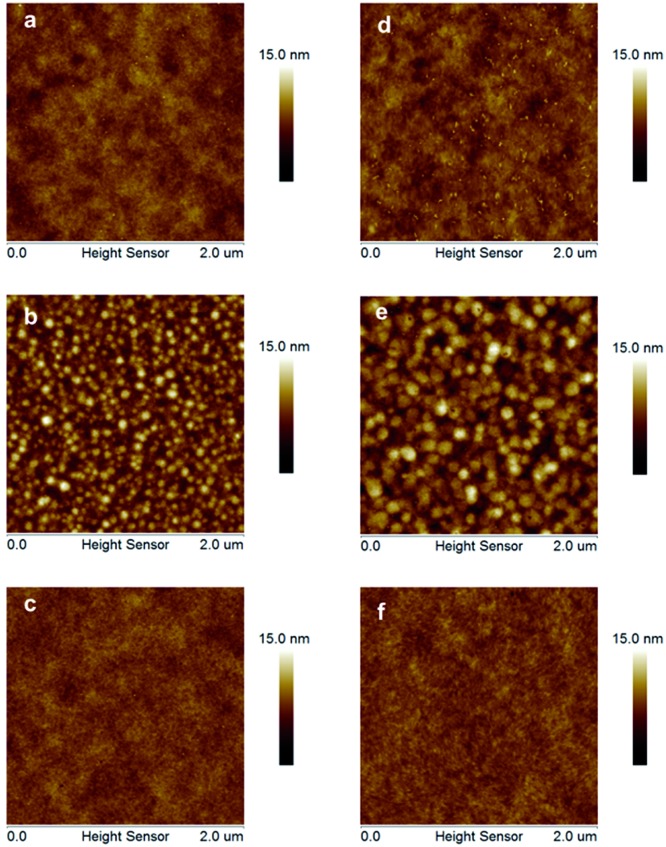
AFM morphology images (2 µm × 2 µm). (a) **PDTPO-IDT** pure film, RMS: 0.88 nm. (b) **PDTPO-IDT** : PC_71_BM blend film without DIO, RMS: 1.62 nm. (c) **PDTPO-IDT** : PC_71_BM blend film with 3% DIO, RMS: 0.68 nm. (d) **PDTPO-IDTT** pure film, RMS: 0.99 nm. (e) **PDTPO-IDTT** : PC_71_BM blend film without DIO, RMS: 1.87 nm. (f) **PDTPO-IDTT** : PC_71_BM blend film with 3% DIO, RMS: 0.72 nm.

The photovoltaic properties of **PDTPO-IDT** and **PDTPO-IDTT** were evaluated in a conventional device structure: ITO/PEDOT:PSS (40 nm)/polymer:PC_71_BM (85 nm)/Ca (20 nm)/Al (100 nm) (Fig. S3†), where ITO (indium tin oxide) was the anode, PEDOT:PSS (poly(3,4-ethylenedioxythiophene):poly(styrenesulfonate)) was used as the hole transporting layer (HTL), PC_71_BM served as the electron acceptor, and Ca/Al worked together as the cathode. The active layers were fabricated by spin-coating a blend solution of **PDTPO-IDT** (or **PDTPO-IDTT**) and PC_71_BM in *o*-DCB (10 mg mL^–1^) with an optimal weight ratio of 1 : 2 at 1600 rounds per minute (rpm), controlling the film thickness at *ca.* 85 nm. 3% (v/v) 1,8-diiodoctane (DIO) was employed as a solution additive to optimize the nanoscale morphology. Characteristic current density–voltage (*J*–*V*) curves of the studied PSCs as well as corresponding external quantum efficiency (EQE) spectra are shown in Fig. 4. The key photovoltaic parameters are summarized in Table 2. PSCs based on **PDTPO-IDT** : PC_71_BM (1 : 2, w/w) and **PDTPO-IDTT** : PC_71_BM (1 : 2, w/w) showed high *V*_oc_ values in the range of 0.94–0.98 V. After adding 3% DIO as additive, the *V*_oc_ values of the two PSCs slightly declined by 0.01 V. However, the *J*_sc_, FF and hole mobility values showed simultaneous enhancement for both PSCs, which is beneficial to achieve higher PCEs. When the donor unit copolymerized with **DTPO** is changed from **IDTT** to **IDT**, the *V*_oc_, *J*_sc_, FF and PCE showed obvious improvement with/without a DIO additive. This can be elucidated from the following aspects: (i) the blend film of **PDTPO-IDT** and PC_71_BM exhibit a broader absorption band than **PDTPO-IDTT**, which is helpful to attain a larger *J*_sc_; (ii) the blend film of **PDTPO-IDT** and PC_71_BM showed a more uniform morphology (Fig. 6c and f) and higher hole mobility than **PDTPO-IDTT** (Fig. 5), which will be discussed below. A solar cell efficiency of 7.33% was achieved in the PSC based on **PDTPO-IDT** accompanied with a high *V*_oc_ of 0.97 V, high FF of 71.5% and a relatively large *J*_sc_ of 10.55 mA cm^–2^. To the best of our knowledge, this PCE value represents the highest value so far for PSCs based on polymers with bandgaps over 2.0 eV (see Table 3). Additionally, an average PCE of 7.17% for **PDTPO-IDT** was obtained from 20 devices, indicating good reproducibility.

**Table 2:** Photovoltaic properties of PSCs based on **PDTPO-IDT** : PC_71_BM (1 : 2, w/w) and **PDTPO-IDTT** : PC_71_BM (1 : 2, w/w) in conventional structures under AM 1.5 G at 100 mW cm^–2^

Polymer	DIO (v/v)	*V*_oc_ (V)	*J*_sc_ (mA cm^–2^)	FF (%)	PCE^*a*^ (%)
**PDTPO-IDT**	0%	0.98	9.85	64.9	6.24 (5.97)
	3%	0.97	10.55	71.5	7.33 (7.17)
**PDTPO-IDTT**	0%	0.95	8.48	59.8	4.83 (4.81)
	3%	0.94	9.14	63.9	5.47 (5.40)

^*a*^The values in parentheses are average efficiencies obtained from 20 devices.

**Table 3:** Comparison of optical bandgap, photovoltaic characteristics and energy levels of polymers with a bandgap over 2.0 eV

Donor	Acceptor	*E* opt g (eV)	PCE (%)	*V*_oc_ (V)	*J*_sc_ (mA cm^–2^)	FF	*E*_HOMO_ ^*a*^ (eV)	*E*_LUMO_ (eV)	Ref.
**PDTPO-IDT**	PC_71_BM	2.05	7.33	0.97	10.55	0.71	–5.32	–3.27	This work
**PDTPO-IDTT**	PC_71_BM	2.04	5.43	0.94	9.14	0.64	–5.31	–3.27	This work
**PDTPO-BDTO** ^*b*^	PC_71_BM	2.02	6.84	0.93	10.4	0.70	–5.38	–3.37	40
**PTADTBTO**	PC_71_BM	2.10	4.64	0.85	11.02	0.59	–5.38	–3.28	48
**PBDTT**	PC_71_BM	2.13	6.12	0.93	11.95	0.55	–5.44	–3.46	47
**PBDT[2F]T**	PC_71_BM	2.1	7.0	0.9	10.7	0.72	–5.29	NA	26
**PIDTT-TzTz**	PC_71_BM	2.0	5.9	0.9	10.41	0.59	–5.24	–3.21	45
**BTT-BTz**	PC_71_BM	2.05	5.06	0.81	10.9	0.57	–5.65^*c*^	–3.60	43
**HMW-P1**	PC_71_BM	2.0	6.52	0.90	12.72	0.57	–5.41	–3.41	49
**PBnDT-FTAZ**	PC_61_BM	2.00	7.10	0.79	12.45	0.72	–5.36	–3.05	25
**PPDT1**	PC_71_BM	2.00	3.28	0.87	8.30	0.45	–5.27	–2.68	41

^*a*^Obtained from cyclic voltammetry.

^*b*^Achieved with an inverted architecture.

^*c*^Obtained from ultraviolet photoelectron spectroscopy.

The external quantum efficiency (EQE) curves of **PDTPO-IDT** : PC_71_BM and **PDTPO-IDTT** : PC_71_BM based devices under optimized conditions are shown in Fig. 4b. Broad EQE spectra are observed in the range of 300 to700 nm, which is extended by dozens of nanometers relative to the UV-vis absorption range at long wavelengths. The PSC based on **PDTPO-IDT** : PC_71_BM (1 : 2, w/w) exhibited an EQE exceeding 60% in range of 370 to 590 nm with the maximum value over 70% at 530 nm. The measured *J*_sc_ of two PSCs were well matched with the corresponding integral value of the EQE spectra within an error of 4%.

### Hole mobility properties and morphology

Hole mobility is an important factor for the performance of PSCs. The hole mobilities of **PDTPO-IDT** and **PDTPO-IDTT** in both pure films and blend films were investigated by employing hole-only devices with the following structure:

ITO/MoO_3_/polymer/MoO_3_/Al for the pure films and ITO/MoO_3_/polymer:PC_71_BM/MoO_3_/Al for the blend films using the space-charge-limited current (SCLC) method. Current–voltage (*I*–*V*) characteristics and SCLC fittings of devices are shown in Fig. 5. In the pure films, **PDTPO-IDT** and **PDTPO-IDTT** exhibit the same hole mobilities of 1.0 × 10^–3^ cm^2^ V^–1^ s^–1^. In the blend films without DIO, the hole mobilities of **PDTPO-IDT** and **PDTPO-IDTT** are 7.6 × 10^–4^ cm^2^ V^–1^ s^–1^ and 6.0 × 10^–4^ cm^2^ V^–1^ s^–1^, respectively. When 3% DIO was added, the hole mobilities are increased to 8.7 × 10^–4^ cm^2^ V^–1^ s^–1^ and 7.6 × 10^–4^ cm^2^ V^–1^ s^–1^, respectively. Compared with the pure films, the presence of PC_71_BM in blend films had a negative effect on the molecular packing and the hole mobility. But the difference of hole mobilities between pure film and blend film are not obvious, indicating that the two polymers have good miscibility with PC_71_BM under the aid of solution annealing by molecular reorganization and repacking, which will contribute to the formation of good phase separation and favorable morphologies. This can also be evident from the atomic force microscopy (AFM) images shown in Fig. 6. The pure films of **PDTPO-IDT** and **PDTPO-IDTT** displayed smooth and uniform morphology with root-mean-square (RMS) surface roughness of 0.875 nm and 0.986 nm, respectively. When PC_71_BM was blended without the 3% DIO additive, it is clearly observed from AFM images (Fig. 6b and e) that many small particles aggregated to form severe phase separations, which is not conducive to charge carrier transport. When 3% DIO was added, the small particles disappeared and the surface morphology returned to a more smooth and uniform state with a RMS roughness of 0.679 nm and 0.719 nm, respectively, indicating that DIO addition played an important role in morphology control.

**PDTPO-IDT** and **PDTPO-IDTT** possess lower HOMO energy levels than **P3HT** (–5.19 eV) by about 0.23 eV. Hence, higher *V*_oc_ values in **PDTPO-IDT** and **PDTPO-IDTT** based PSCs could be anticipated compared to that of the **P3HT** based PSC, as the *V*_oc_ value and the difference in energy between the HOMO of the electron donor and the LUMO of the electron acceptor are positively and linearly correlated.^50^,^51^ Interestingly, the HOMO energy level of **PDTPO-IDT** is higher than that of **PDTPO-BDTO** (–5.38 eV) by 0.06 eV,^40^ however, the **PDTPO-IDT** based PSC achieved a higher *V*_oc_ (Table 3). This cannot be simply explained by using the above-mentioned principle. A more accurate *V*_oc_ value can be calculated using the following equation:^46^1
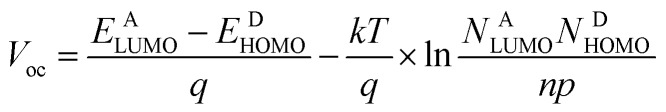
where *E*ALUMO is the LUMO energy level of the acceptor (A) and *E*DHOMO is the HOMO energy level of the donor (D). *N*ALUMO is the density of states of the LUMO of the acceptor and *N*DHOMO is the density of states of the HOMO of the donor. *n* is the density of electrons of the acceptor and *p* is the density of holes of the donor. *q* is the elementary charge, *k* is the Boltzmann constant, and *T* is the absolute temperature. After replacing **BDTO** with ladder-type **IDT**, the hole mobility of the blend film with **PDTPO-IDT** is nearly sevenfold higher than that of **PDTPO-BDTO** (1.25 × 10^–4^ cm^2^ V^–1^ s^–1^), which means that larger hole and electron concentration gradients would be built up in the blend film, following that the values of *n* and *p* accordingly increase. Meanwhile, nearly equal *N*ALUMO*N*DHOMO values can be predicted in the two PSCs, indicated by the approximately equivalent *J*_sc_ of the two PSCs. A lower loss of *V*_oc_ can be calculated from the second term of eqn (1) for PSC with **PDTPO-IDT**. According to the equation, although the first term of eqn (1) is somewhat smaller for *V*_oc_ of the **PDTPO-IDT** based PSC, it can still yield a higher *V*_oc_ over the **PDTPO-BDTO** based PSC.

## Conclusion

In summary, we developed two new wide bandgap polymers through the combination of ladder-type **IDT**/**IDTT** units with the planar **DTPO** unit. Both of the two polymers showed wide optical bandgaps exceeding 2.0 eV and deep HOMO energy levels of *ca.* –5.32 eV. PSCs based on **PDTPO-IDT** with bandgaps of up to 2.05 eV achieved a remarkable *V*_oc_ of 0.97 V and a high efficiency of 7.33%. **PDTPO-IDTT** based PSCs showed a moderate PCE of 5.47%. The small torsion angle, high hole mobility and uniform morphology contribute to the high performance of the **PDTPO-IDT** based PSCs. We believe that the wide optical bandgap and excellent device performance of **PDTPO-IDT** will make it a promising candidate for tandem and ternary organic solar cells.

## Experimental section

### General information

All chemicals were purchased from commercial sources and used without further purification unless otherwise stated. The solvents were dried using standard procedures when necessary. 2,8-Bis(trimethyltin)-indacenodithiophene (**IDT-2Sn(Me)_3_**) and 2,10-bis(trimethyltin)-indacenodithieno[3,2-*b*]thiophene (**IDTT-2Sn(Me)_3_**) were purchased from commercial sources with a purity of 98%. Monomer 2,6-dibromo-4-(2-octyldodecyl)-dithieno[3,2-*b*:2′,3′-*d*]pyridin-5(4*H*)-one (**DPTO-2Br**) was synthesized according to literature methods.^40^ UV-vis spectra were measured using a Shimadzu UV-2500 recording spectrophotometer. ^1^H and ^13^C NMR spectra were collected on a MERCURY-VX300 spectrometer operating at 300 MHz using deuterated chloroform referenced to tetramethylsilane (TMS). Electron ionization mass spectrometry (EI-MS) was recorded on a VJ-ZAB-3F-mass spectrometer. Elemental analysis (EA) was carried out on a Vario EL-III microanalyzer to identify the content of carbon, hydrogen, nitrogen and sulfur. Gel permeation chromatography (GPC) was measured on a Waters 2690 D system using a refractive detector and tetrahydrofuran (THF) as the eluent. Cyclic voltammetry (CV) measurements of polymer films were conducted on a CHI voltammetric analyzer in acetonitrile solution with 0.1 M tetrabutylammonium hexafluorophosphate (*n*-Bu_4_NPF_6_) as supporting electrolyte at room temperature by using a scan rate of 100 mV s^–1^ and a conventional three-electrode configuration consisting of a platinum working electrode with 2 mm diameter, a platinum wire counter electrode and a Ag/AgCl wire reference electrode. Thermogravimetric analysis (TGA) was performed on a Perkin Elmer Pyris under a nitrogen atmosphere at a heating rate of 10 °C min^–1^. The temperature of degradation (*T*_d_) was correlated with 5% weight loss. Atomic force microscopy (AFM) images were obtained by using a NanoMan VS microscope in tapping-mode.

### Polymer synthesis

#### **PDTPO-IDT**

2,8-Bis(trimethyltin)-indacenodithiophene (**IDT-2Sn(Me)_3_**) (246.9 mg, 0.2 mmol), **DTPO-2Br** (129.1 mg, 0.2 mmol) and anhydrous toluene (10 mL) were added to a Schlenk tube. The tube was purged with argon for 30 minutes to remove the oxygen, and then the tetrakis(triphenylphosphine)palladium(0) (11.5 mg, 5 mmol%) was added. After flushing with argon for another 30 minutes, the tube was sealed and the reaction mixture was stirred at 110 °C for 48 h. 4-Bromotoluene (0.2 mL) was added to complete the end-capping by stirring at 110 °C overnight. The mixture was cooled to room temperature and poured into methanol (150 mL). After precipitation in methanol, the precipitate was filtered through a Soxhlet thimble, which was then subjected to Soxhlet extraction with acetone for 12 h, hexane for 12 h and chloroform for 12 h, successively. The chloroform fraction was concentrated by rotary evaporation and precipitated in methanol again. The polymer was collected and dried under vacuum for 24 h as a deep red power with a yield of 71% (197 mg). GPC: *M*_n_ = 23.9 kDa, PDI = 1.7. ^1^H NMR (300 MHz, CDCl_3_, *δ* (ppm)): 7.71 (br, s, 1H), 7.42 (m, 3H), 7.12–7.19 (br, 16H), 5.35 (br, s, 2H), 2.57 (br, s, 8H), 2.23 (br, s, 1H), 2.01 (br, s, 2H), 1.27 (m, 64H), 0.88 (m, 18H). Anal. calcd (%) for (C_93_H_115_NOS_4_)_*n*_: C, 80.29; H, 8.33; N, 1.01; S, 9.22; found: C, 80.18; H, 8.27; N, 0.99; S, 9.28.

#### **PDTPO-IDTT**

**PDTPO-IDTT** was synthesized according to the same procedure as **PDTPO-IDT**, except using the monomer 2,10-bis(trimethyltin)-indacenodithieno[3,2-*b*]thiophene (**IDTT-2Sn(Me)_3_**) (269.3 mg, 0.2 mmol), and a deep red solid was obtained with a yield of 92% (278 mg). GPC: *M*_n_ = 30.2 kDa, PDI = 2.2. ^1^H NMR (300 MHz, CDCl_3_, *δ* (ppm)): 7.70 (br, s, 1H), 7.52 (m, 2H), 7.42 (s, 1H), 7.13–7.19 (br, 16H), 2.57 (br, s, 8H), 1.29 (m, 64H), 0.86 (m, 18H). Anal. calcd (%) for (C_97_H_115_NOS_6_)_*n*_: C, 77.50; H, 7.71; N, 0.93; S, 12.80; found: C, 77.52; H, 7.58; N, 0.92; S, 12.96.

### Fabrication and characterization of solar cells

Polymer solar cells (PSCs) with a conventional device structure of ITO/PEDOT:PSS/polymer:PC_71_BM/Ca/Al were fabricated. The patterned ITO-coated glass was scrubbed with detergent and then cleaned in an ultrasonic bath by using deionized water, acetone, and isopropyl alcohol sequentially, and dried overnight in an oven before use. Then, a PEDOT:PSS (Heraeus Clevios P VP A 4083) layer was spin-coated onto the ITO with a thickness of 40 nm, and then dried at 150 °C under air conditions for 10 min. Next, the photoactive layer with an optimal thickness of 85 nm was prepared by spin casting the mixed solution of **PDTPO-IDT** (or **PDTPO-IDTT**) and PC_71_BM in *o*-dichlorobenzene (the concentration of **PDTPO-IDT** or **PDTPO-IDTT** is 10 mg mL^–1^ for all blend films) with different weight ratio and DIO concentration at 1600 rpm for 40 s on the top of the PEDOT:PSS layer. After that, methanol was spin-coated atop the **PDTPO-IDT** (or **PDTPO-IDTT**):PC_71_BM blend layer at 2500 rpm for 30 s before the deposition of the cathode. Finally, a 20 nm Ca and 100 nm Al layer were subsequently evaporated onto the active layer through a shadow mask at a vacuum pressure of ≈5 × 10^–5^ Pa to form the top electrode. The overlapping proportion between cathode and anode was 4.5 mm^2^. In order to accurately measure the performance of PSCs, an aperture with the area of 3.14 mm^2^ was used. The current–voltage (*J*–*V*) characteristics were measured using a Keithley 2400 Source Measure Unit. The solar cell performance was tested under an irradiation intensity of 100 mW cm^–2^ measured by a calibrated silicon solar cell and a readout meter (Model 91150V, Newport) using an Air Mass 1.5 Global (AM 1.5 G) solar simulator (Class AAA solar simulator, Model 94063A, Oriel). The EQE spectra were measured using a QEX10 Solar Cell IPCE measurement system (PV measurement, Inc). All the fabrication processes were carried out inside a dry glovebox filled with nitrogen, except for the spin-coating of PEDOT:PSS.

## Supplementary Material

Supplementary information
