# Supplementary material for: Dithieno[3,2-b:2′,3′-d]pyridin-5(4H)-one based D–A type copolymers with wide bandgaps of up to 2.05 eV to achieve solar cell efficiencies of up to 7.33%
Source: Chem Sci. 2016 Jun 10;7(9):6167–75. doi: 10.1039/c6sc01791f (PMC6022258; doi:10.1039/c6sc01791f)
Supplement: Supplementary file 1 [file SC-007-C6SC01791F-s001.pdf]

Electronic Supplementary Material (ESI) for Chemical Science.
This journal is © The Royal Society of Chemistry 2016

Electronic Supplementary Information

Dithieno[3,2-*b*:2',3'-*d*]pyridin-5(4*H*)-one based D-A type copolymers with wide bandgap up to 2.05 eV to achieve solar cell efficiency up to 7.33%

Wei Gao,†[a] Tao Liu,†[b] Minghui Hao,[a] Kailong Wu,[a] Chen zhang,[a] Yanming Sun*[b] and Chuluo Yang*[a]

[a] Hubei Collaborative Innovation Center for Advanced Organic Chemical Materials, Hubei Key Lab on Organic and Polymeric Optoelectronic Materials, Department of Chemistry, Wuhan University, Wuhan 40072, China. E-mail: clyang@whu. edu. cn

[b] Heeger Beijing Research and Development Center, School of Chemistry and Environment, Beihang University, Beijing 100191, P. R. China. E-mail: sunym@buaa. edu. cn

† The two authors contributed equally to this work

Table of Contents

1. TGA plots of PDTPO-IDT and PDTPO-IDTT

2. Electron density distribution of HOMO and LUMO levels calculated from DFT

3. Device structure of studied PSCs

4. Absorption spectra of blend films with 3 % DIO

5. ^{13}C NMR of DTPO unit and ^{1}H NMR spectra of PDTPO-IDT and PDTPO-IDTT

6. Corresponding HOMO and LUMO levels obtaining from DFT

7. Mean value and mean square error of each key parameters (V_{oc}, J_{sc}, FF, PCE) of PSCs

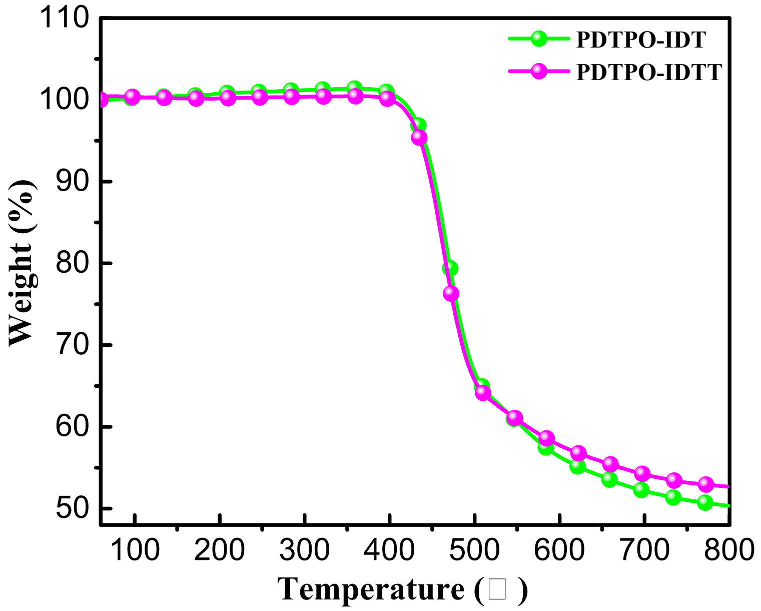

Fig. S1. TGA plots of PDTPO-IDT and PDTPO-IDTT with a heating rate of 10 ^{o}C/min under inert atmosphere.

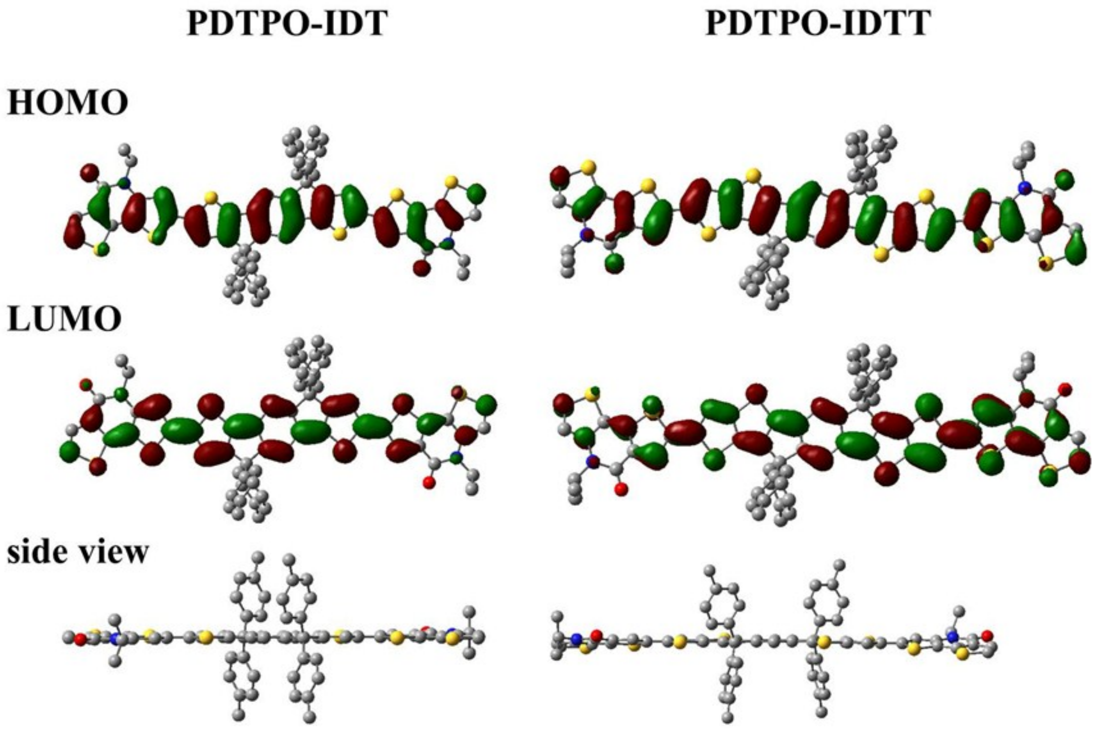

Fig. S2. Electron density distribution of HOMO and LUMO energy levels and side view of PDTPO-IDT and PDTPO-IDTT calculated from the level of B3LYP/6-31G*.

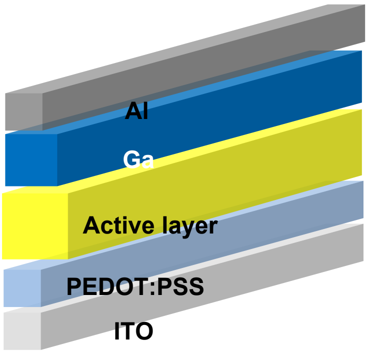

Fig. S3. Devices structure of PSC based on PDTPO-IDT and PDTPO-IDTT.

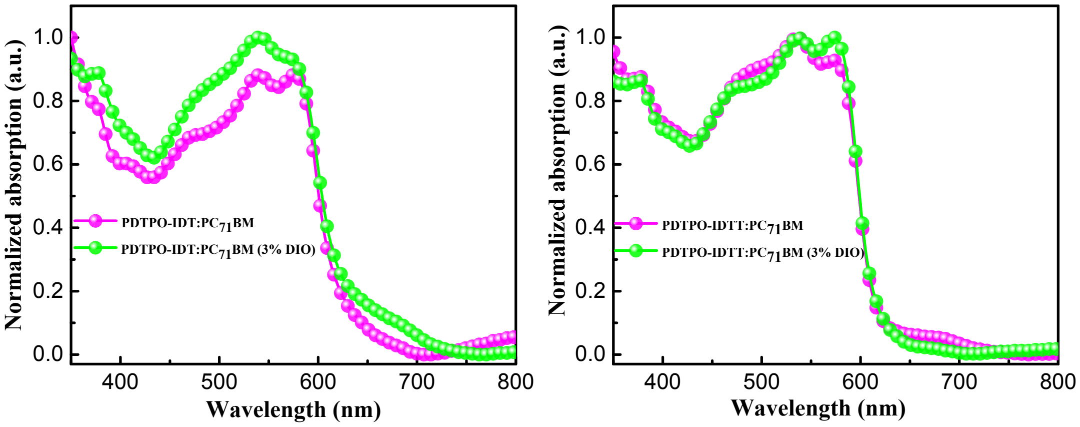

Fig. S4. Normalized UV-vis absorption spectra of blend films with or without DIO for PDTPO-IDT (a) and PDTPO-IDTT (b).

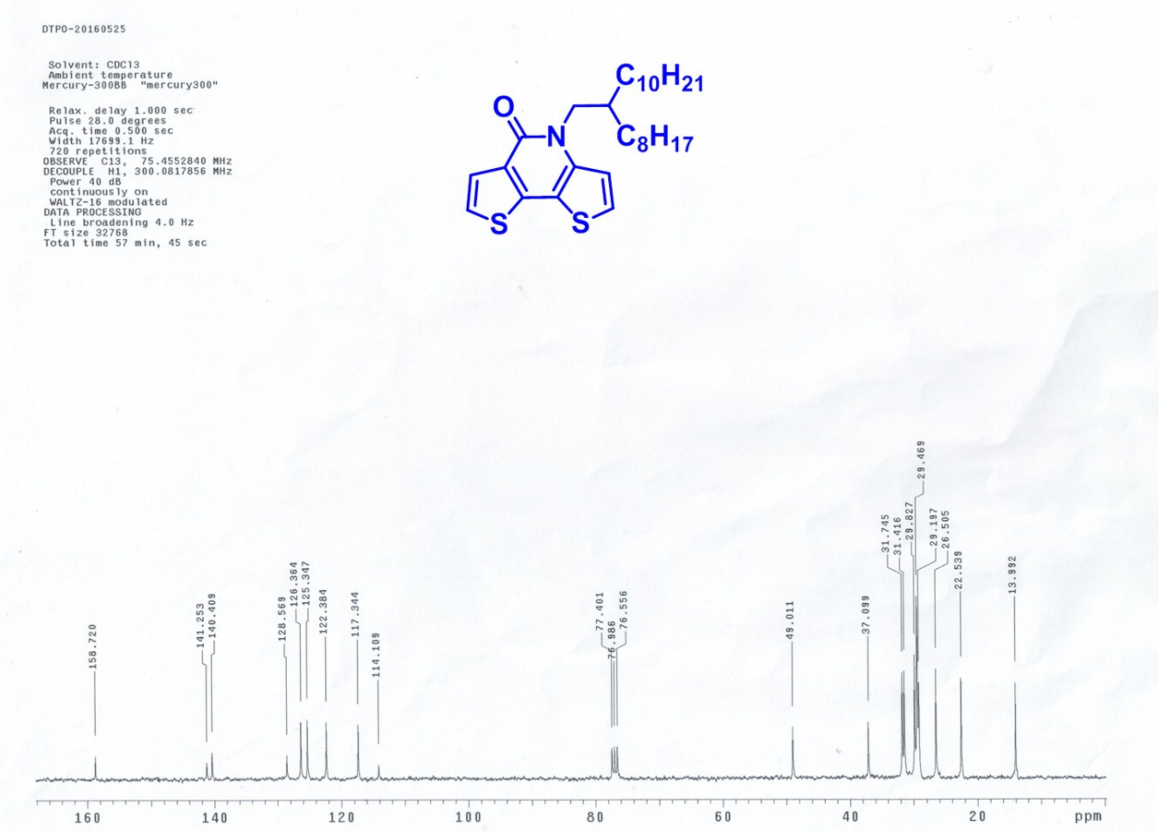

Fig. S5. ^{13}C NMR spectrum (300 MHz) of DTPO unit in $CDCl_3$.

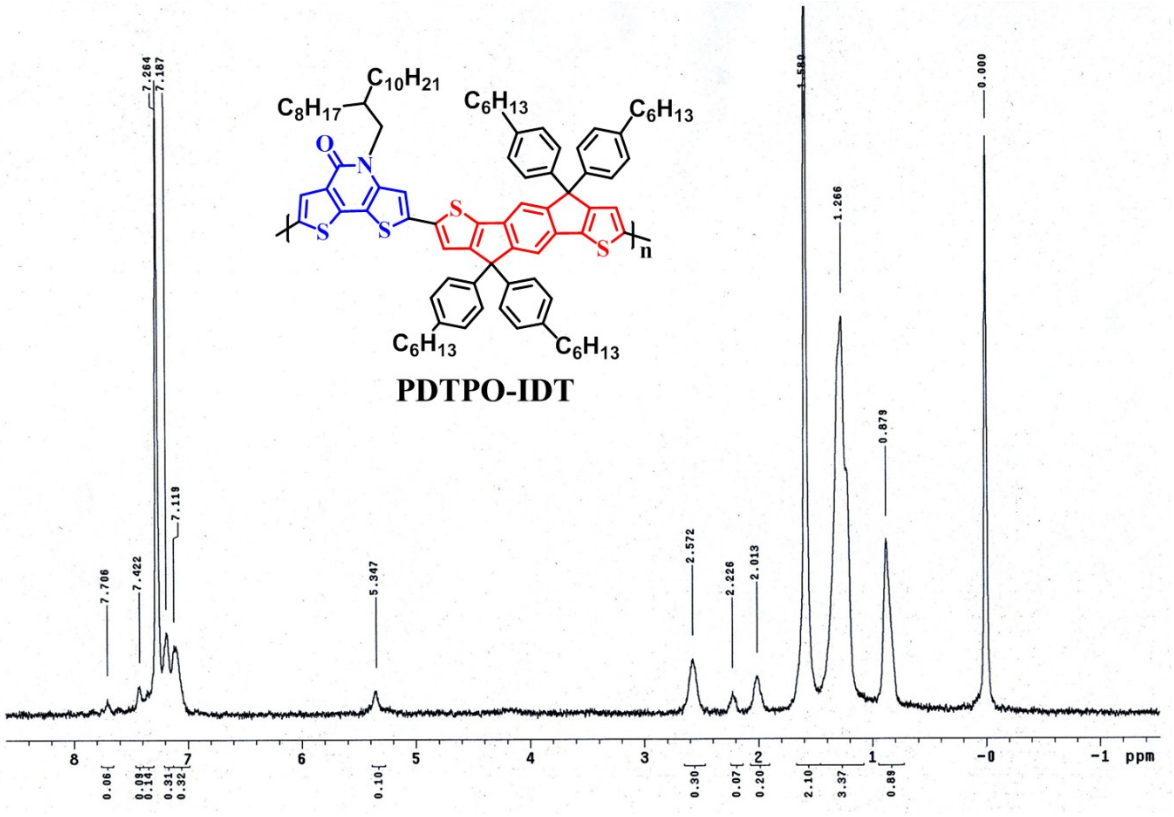

Fig. S6. ^{1}H NMR spectrum (300 MHz) of PDTPO-IDT in $CDCl_3$.

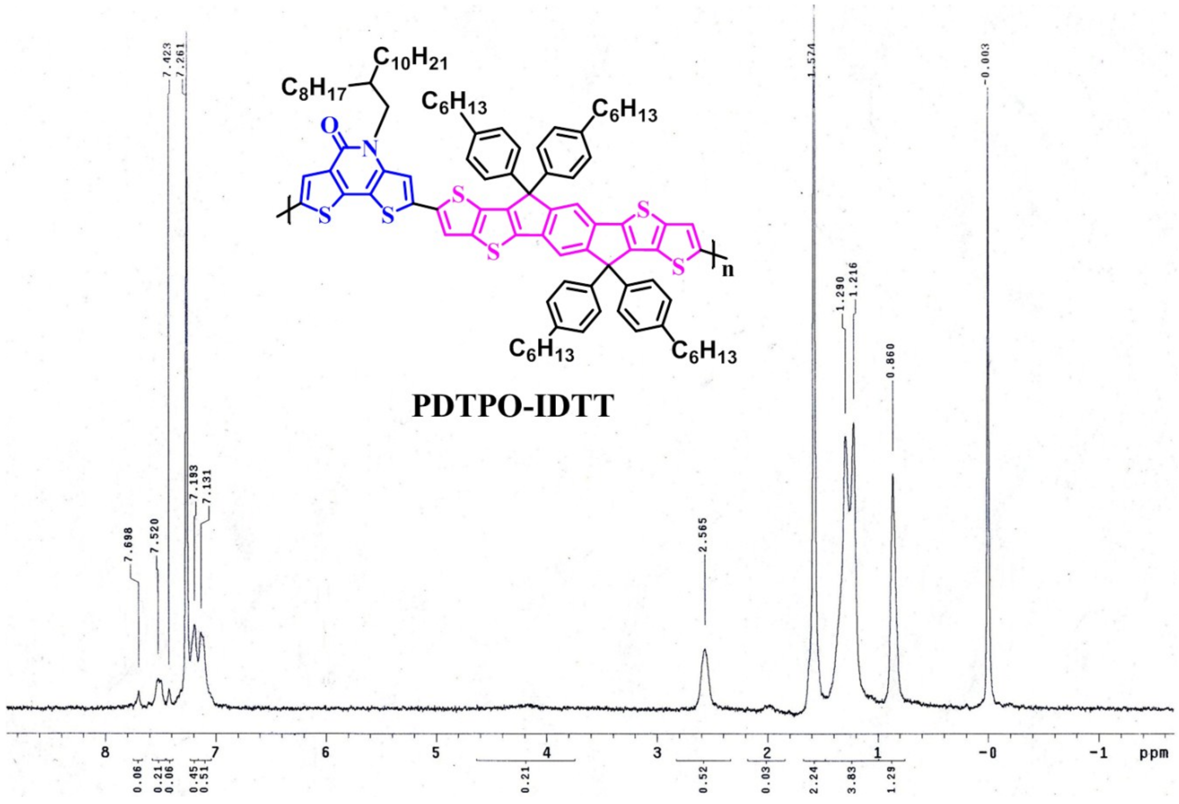

Fig. S7. ^{1}H NMR spectrum (300 MHz) of PDTPO-IDTT in $CDCl_3$.

Table S1. HOMO and LUMO energy levels obtaining from theory calculation

Polymer	HOMO (eV)	LUMO (eV)	E_g^{opt} (eV)
**PDTPO-IDT**	-4.76	-2.17	2.59
**PDTPO-IDTT**	-4.74	-2.18	2.56

Table S2. Mean value and mean square error of each key parameters (V_{oc}, J_{sc}, FF, PCE) of PSCs from 20 devices.

Polymer	DIO (v/v)	V_{oc} (V)	J_{sc} (mA/cm^2)	FF (%)	PCE (%)	PCE$_{max}$ (%)
**PDTPO-IDT**	0%	0.966 ± 0.008	9.715 ± 0.10	0.636 ± 0.009	5.969 ± 0.184	6.239
**PDTPO-IDT**	3%	0.964 ± 0.006	10.44 ± 0.16	0.712 ± 0.005	7.167 ± 0.165	7.332
**PDTPO-IDTT**	0%	0.947 ± 0.004	8.524 ± 0.10	0.596 ± 0.005	4.812 ± 0.045	4.826
**PDTPO-IDTT**	3%	0.938 ± 0.005	9.200 ± 0.22	0.627 ± 0.014	5.403 ± 0.064	5.468
